# Association of Oxidative Balance Score With All-Cause and Cardiovascular Mortality Among Hypertensive Adults

**DOI:** 10.31083/RCM37415

**Published:** 2025-08-15

**Authors:** Bing Hu, Xin He, Yanxiang Sun, Tong Liu, Fei Li, Li Feng, Yuli Huang

**Affiliations:** ^1^Department of Cardiology, The Eighth Affiliated Hospital, Southern Medical University (The First People’s Hospital of Shunde, Foshan), 528300 Foshan, Guangdong, China; ^2^Department of Cardiology, Zhongshan City People’s Hospital, 528400 Zhongshan, Guangdong, China; ^3^Department of Hematology, Zhongshan City People’s Hospital, 528400 Zhongshan, Guangdong, China; ^4^Department of Food Policy, George Institute for Global Health, Faculty of Medicine, University of New South Wales, Sydney, NSW 2052, Australia; ^5^Guangdong Engineering Technology Research Center of Metabolic Disorders Interdisciplinary Precision Prevention and Digital Healthcare, 528300 Foshan, Guangdong, China; ^6^Foshan Precision Medicine Engineering Center for Cardiovascular Diseases, 528300 Foshan, Guangdong, China

**Keywords:** oxidative balance score, oxidative stress, hypertension, cardiovascular, mortality

## Abstract

**Background:**

The Oxidative Balance Score (OBS) is a new measure for assessing systemic oxidative stress, where higher scores indicate increased exposure to antioxidants. However, the relationship between the OBS and mortality in individuals with hypertension remains unclear.

**Methods:**

This study evaluated 8151 hypertensive individuals from the National Health and Nutrition Examination Survey (NHANES) (2001–2018), utilizing data from the National Death Index, tracked through December 31, 2019. The association between OBS and mortality (cardiovascular and all-cause) was examined using multivariable Cox regression models.

**Results:**

During a median follow-up of 9.7 years, which included 1692 deaths (461 of which were cardiovascular), multivariable Cox regression showed the highest quartile of OBS had significantly lower rates of all-cause mortality (hazard ratio (HR) 0.761, 95% CI: 0.635–0.912) and cardiovascular mortality (HR 0.553, 95% CI: 0.388–0.788) compared to those in the lowest quartile. An increase of one unit in the OBS was associated with a 1.9% reduction in all-cause mortality risk and a 3.7% reduction in cardiovascular mortality risk. This relationship remained consistent across various subgroup analyses, and spline regression supported a linear inverse trend.

**Conclusions:**

For adults with hypertension, an elevated OBS is independently associated with a lower risk of mortality both from all-cause and cardiovascular diseases, suggesting that higher antioxidant levels may be protective.

## 1. Introduction 

Hypertension is a widespread and significant public health issue that affects over one billion individuals globally [1]. It is a major contributor to cardiovascular disease, including heart failure, coronary artery disease, chronic kidney conditions, and stroke, which significantly raises global morbidity and mortality rates [2, 3]. Epidemiologically, hypertension is associated with a heavy burden on healthcare systems, as it often leads to life-threatening complications when left uncontrolled [4]. Oxidative stress refers to an imbalance between the production of reactive oxygen species (ROS) and the body’s ability to neutralize them through antioxidant systems [5]. This imbalance plays a crucial role in developing hypertension and its associated complications [6, 7, 8]. This imbalance contributes to endothelial dysfunction [9], arterial stiffness, and vascular inflammation [10, 11], all of which promote the development and progression of hypertension. A comprehensive understanding of the mechanisms underlying oxidative stress is essential for elucidating its role in hypertension and for the development of targeted therapeutic strategies.

The Oxidative Balance Score (OBS) is an innovative measure designed to assess overall oxidative stress by combining pro-oxidant and antioxidant factors [12]. This metric takes into account various aspects, such as dietary intake, lifestyle behaviors, and specific biochemical indicators to determine an individual’s oxidative profile [13]. Higher OBS values generally indicate greater antioxidant exposure and a healthier redox state, while lower values suggest heightened oxidative burden due to increased pro-oxidant exposure [14]. Although oxidative stress has been extensively studied in chronic diseases [15, 16, 17, 18], few studies link OBS to mortality in those with hypertension. This research addresses that gap by investigating how OBS relates to mortality among hypertensive patients. The findings could enhance our understanding of oxidative balance, guide future interventions, improve hypertension management, and ultimately lead to better patient outcomes.

## 2. Methods

### 2.1 Study Population

**Fig. 1.**
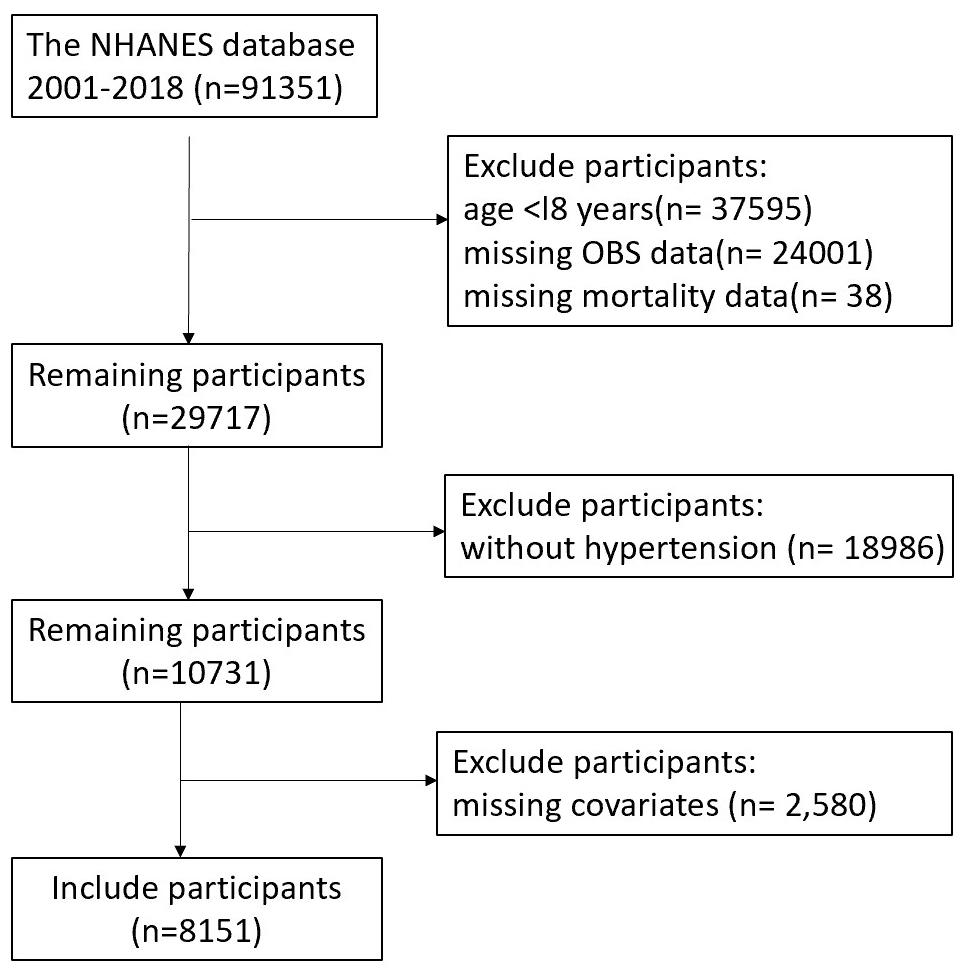
**Participant selection flowchart**. NHANES, National Health and Nutrition Examination Survey; OBS, Oxidative Balance Score.

This study analyzed data from 8151 hypertensive adults participating in the National Health and Nutrition Examination Survey (NHANES) between 2001 and 2018. Inclusion criteria were: (1) age ≥18 years; (2) diagnosis of hypertension, defined as systolic blood pressure ≥140 mmHg, diastolic blood pressure ≥90 mmHg, or current use of antihypertensive medication. Participants lacking essential information, including the OBS, covariates, or mortality data, were excluded from this analysis (Fig. 1). The National Centre for Health Statistics (NCHS) Ethics Review Board approved the study, and all participants provided informed consent.

### 2.2 Evaluation of OBS

The OBS integrates 16 dietary elements and four lifestyle variables, including antioxidants and prooxidants [17]. The nutritional elements considered include carotenoids, fiber, vitamins C, E, B6, B12, folate, riboflavin, niacin, total fat, calcium, copper, magnesium, zinc, selenium, and iron [16]. Lifestyle variables evaluated were body mass index (BMI), smoking status, alcohol intake, and physical activity. Smoking intensity was assessed using cotinine levels. The pro-oxidant variables included iron, alcohol, cotinine, BMI, and total fat, while the remaining were considered antioxidant factors.

In our approach, each component was equally weighted. For scoring, each variable was divided into gender-specific tertiles. Antioxidants received a score ranging from 0 to 2, with the highest tertile receiving 2 points and the lowest receiving 0. Conversely, pro-oxidants were scored in reverse order. The total OBS ranged from 3 to 36, with higher values reflecting a more favorable oxidative balance [19]. It is important to note that our scoring method did not incorporate potential interactions between the dietary and lifestyle factors. Detailed scoring procedures are provided in **Supplementary Table 1**.

### 2.3 Definition of Hypertension

Hypertension is defined through self-reported diagnosis, ongoing blood pressure medication use, or elevated blood pressure measurements. Blood pressure was measured three times to ensure accuracy, with hypertension categorized by systolic pressure reaching or exceeding 140 mmHg and/or diastolic pressure at or above 90 mmHg.

### 2.4 Determination of Mortality

Mortality data for NHANES participants were tracked until December 31, 2019, using the Linked National Death Index. Causes of death were classified according to the International Classification of Diseases, Tenth Revision (ICD-10) codes, with codes I00-I09, I11, I13, and I20-I51 identifying cardiovascular deaths.

### 2.5 Covariates

To address potential confounding factors, the study incorporated various covariates. Demographic data included gender, age, and ethnicity (Mexican American, Non-Hispanic White, Non-Hispanic Black, Other), education level (less than high school, high school graduate/equivalent, above high school), and marital status (partnered [married/cohabiting] or single [never married/divorced/widowed]). Health behaviors included alcohol use (current, former, non-drinkers) and smoking status (current, former, never). Diabetes was identified through self-reported diagnosis, medication use, or fasting glucose levels of 126 mg/dL or higher. Hyperlipidemia was identified based on total cholesterol levels ≥240 mg/dL, current use of lipid-lowering medication, or a self-reported diagnosis. The poverty-to-income ratio (PIR) was used to assess socioeconomic status.

### 2.6 Statistical Analysis

Following categorization into four groups based on OBS quartiles, Continuous variables were expressed as mean ± standard deviation (SD) or median (interquartile range, IQR), depending on their distribution, while categorical variables were presented as counts and percentages. The Kolmogorov–Smirnov test assessed the normality of continuous variables. The Kruskal–Wallis test was employed to compare means across OBS quartiles for non-normally distributed variables. Categorical variables were compared using the chi-square test.

A stepwise approach assessed the relationship between OBS and mortality risk through Cox proportional hazard regression. An initial unadjusted model (Model 1), followed by progressively adjusted models: Model 2 accounted for age, sex, and race/ethnicity, while Model 3 further included education, marital status, PIR, smoking, alcohol use, and diabetes status. Kaplan-Meier curves and log-rank tests were applied for preliminary survival comparisons. To visualize the dose-response pattern, restricted cubic splines (RCS) were integrated into the fully adjusted Cox model. Consistency across different populations was examined via stratified analyses and tests for interaction. Analyses were conducted in R (version 4.3.3, The R Foundation for Statistical Computing, Vienna, Austria), setting statistical significance at a two-sided *p* < 0.05.

## 3. Results

### 3.1 Baseline Characteristics

Table 1 presents the baseline characteristics of the 8151 hypertensive study participants (54.5% male, average age 57.9 years). The mean OBS of 20, and a range spanning 4 to 36 across all participants. The study population was divided into four quartiles: Q1 (4–14), Q2 (15–20), Q3 (21–26), and Q4 (27–36). A comparison between the lowest (Q1) and highest (Q4) quartiles revealed distinct characteristics: Q1 participants were more likely to be non-Hispanic Black, possess lower educational attainment, and exhibit higher rates of both smoking and diabetes mellitus. Moreover, they were less likely to be married and had elevated PIR.

**Table 1.** **Participant baseline characteristics are distributed across OBS quartiles**.

Variable		Q1 (OBS4–14 n = 2098)	Q2 (OBS15–20 n = 2151)	Q3 (OBS21–26 n = 2209)	Q4 (OBS27–36 n = 1693)	*p*-value
Age (years)		55 (47–71)	55 (47–70)	55 (47–70)	56 (47–70)	0.222
BMI (kg/m^2^)		29.86 (26.31–34.01)	29.80 (26.30–34.22)	29.10 (25.70–33.80)	28.10 (24.80–32.80)	<0.001
Sex						<0.001
	Female	863 (41.13%)	971 (45.14%)	1065 (48.21%)	807 (47.67%)	
	Male	1235 (58.87%)	1180 (54.86%)	1144 (51.79%)	886 (52.33%)	
Race						<0.001
	Mexican American	270 (12.87%)	244 (11.34%)	261 (11.82%)	218 (12.88%)	
	Non-Hispanic Black	649 (30.93%)	534 (24.83%)	422 (19.10%)	269 (15.89%)	
	Non-Hispanic White	956 (45.57%)	1118 (51.98%)	1221 (55.27%)	983 (58.06%)	
	Other Hispanic	125 (5.96%)	147 (6.83%)	141 (6.38%)	92 (5.43%)	
	Other race	98 (4.67%)	108 (5.02%)	164 (7.42%)	131 (7.74%)	
Education						<0.001
	Above high school	876 (41.75%)	1056 (49.09%)	1270 (57.49%)	1071 (63.26%)	
	Below high school	648 (30.89%)	505 (23.48%)	402 (18.20%)	271 (16.01%)	
	High school or equivalent	574 (27.36%)	590 (27.43%)	537 (24.31%)	351 (20.73%)	
Marital						<0.001
	Coupled	1292 (61.58%)	1360 (63.23%)	1416 (64.10%)	1152 (68.04%)	
	Singled	806 (38.42%)	791 (36.77%)	793 (35.90%)	541 (31.96%)	
Smoke						<0.001
	No	876 (41.75%)	1034 (48.07%)	1133 (51.29%)	957 (56.53%)	
	Yes	1222 (58.25%)	1117 (51.93%)	1076 (48.71%)	736 (43.47%)	
Alcohol user						0.610
	No	270 (12.87%)	284 (13.20%)	270 (12.22%)	202 (11.93%)	
	Yes	1828 (87.13%)	1867 (86.80%)	1939 (87.78%)	1491 (88.07%)	
DM status						<0.001
	DM	603 (28.74%)	563 (26.17%)	545 (24.67%)	367 (21.68%)	
	No	1314 (62.63%)	1384 (64.34%)	1427 (64.60%)	1152 (68.04%)	
	Pre-DM	181 (8.63%)	204 (9.48%)	237 (10.73%)	174 (10.28%)	
Hyperlipidemia						0.036
	No	383 (18.26%)	374 (17.39%)	389 (17.61%)	351 (20.73%)	
	Yes	1715 (81.74%)	1777 (82.61%)	1820 (82.39%)	1342 (79.27%)	
PIR-Category						<0.0001
	PIR >3.0	640 (30.51%)	841 (39.10%)	1005 (45.50%)	868 (51.27%)	
	PIR ≤1.0	470 (22.40%)	364 (16.92%)	319 (14.44%)	188 (11.10%)	
	PIR 1.0–3.0	988 (47.09%)	946 (43.98%)	885 (40.06%)	637 (37.63%)	

OBS, oxidative balance score; BMI, body mass index; DM, diabetes mellitus; PIR, poverty-to-income ratio.

### 3.2 Kaplan-Meier Analysis of OBS and Mortality 

**Fig. 2.**
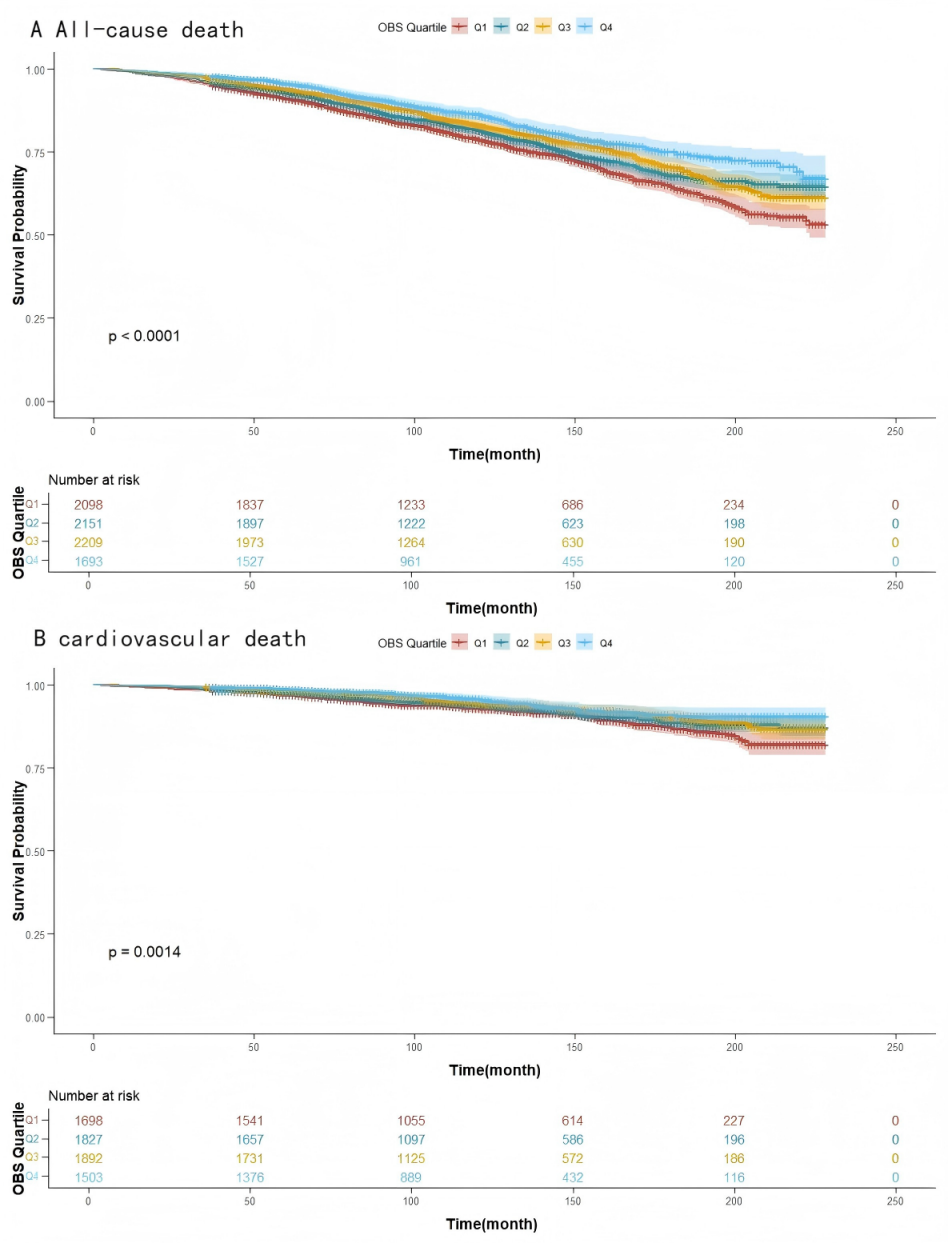
**Kaplan-Meier for mortality**. (A) All-cause mortality survival. (B) Cardiovascular death survival. Q1–Q4, quartiles 1–4.

With a median follow-up of 9.7 years, 1692 deaths (20.8%) were recorded among the 8151 participants, including 461 cardiovascular deaths (27.2% of the total). Kaplan-Meier analysis highlighted significant survival differences based on OBS quartiles. All-cause mortality analysis showed optimal survival in Q4 and the poorest survival in Q1 (Fig. 2A, log-rank *p* < 0.0001). Similarly, for cardiovascular mortality, Q4 demonstrated superior survival compared to the lower quartiles (Fig. 2B, log-rank *p* < 0.001).

### 3.3 Multivariable Cox Analysis: OBS and Mortality Hazard 

The association between OBS and mortality risk, assessed via multivariable Cox analysis, is summarized in Table 2. Specifically, for each additional unit of OBS, there is a 1.9% reduction in the risk of all-cause mortality (Model 3: hazard ratio (HR) 0.981, 95% CI: 0.972–0.991, *p* < 0.001). Analysis by OBS quartiles revealed a significant inverse trend with all-cause mortality (*p* for trend = 0.003), highlighting a substantially lower mortality risk for participants in the highest quartile compared to the lowest (HR 0.761, 95% CI: 0.635–0.912, *p* = 0.003).

**Table 2.** **Adjusted Cox models for OBS and mortality risk**.

OBS		Model 1		Model 2		Model 3	
		HR (95% CI)	*p* value	HR (95% CI)	*p* value	HR (95% CI)	*p* value
All-cause death							
Continuous		0.971 (0.962, 0.980)	<0.001	0.968 (0.959, 0.977)	<0.001	0.981 (0.972, 0.991)	<0.001
Quartiles							
	Q1	Reference		Reference		Reference	
	Q2	0.829 (0.706, 0.974)	0.023	0.853 (0.720, 1.010)	0.065	0.980 (0.833, 1.153)	0.812
	Q3	0.714 (0.596, 0.856)	<0.001	0.718 (0.607, 0.849)	<0.001	0.899 (0.763, 1.061)	0.209
	Q4	0.565 (0.465, 0.686)	<0.001	0.567 (0.474, 0.679)	< 0.001	0.761 (0.635, 0.912)	0.003
	*p* for trend		<0.001		<0.001		0.003
Cardiovascular death							
Continuous		0.966 (0.950, 0.983)	<0.001	0.959 (0.94, 0.975)	<0.001	0.963 (0.947, 0.980)	<0.001
Quartiles							
	Q1	Reference		Reference		Reference	
	Q2	0.890 (0.664, 1.192)	0.434	0.888 (0.668, 1.181)	0.417	0.923 (0.693, 1.230)	0.585
	Q3	0.620 (0.456, 0.842)	0.002	0.608 (0.449, 0.823)	0.001	0.648 (0.470, 0.893)	0.008
	Q4	0.541 (0.382, 0.767)	<0.001	0.506 (0.357, 0.717)	<0.001	0.553 (0.388, 0.788)	0.001
	*p* for trend		<0.001		<0.001		<0.001

Model 1, unadjusted; Model 2, adjusted for age, sex, and race; Model 3, further adjusted for education, marital status, poverty-to-income ratio, drinking, smoking, and diabetes. HR, hazard ratio.

OBS also demonstrated a significant inverse correlation with cardiovascular mortality. As a continuous measure, higher OBS scores predicted a lower risk of cardiovascular death (Model 3: HR 0.963, 95% CI: 0.947–0.980, *p* < 0.001). Analysis by quartiles confirmed this negative association, showing a significant dose-response pattern (*p* for trend < 0.001). Participants in the highest OBS quartile had a 44.7% reduced risk of cardiovascular mortality relative to those with the lowest quartile (HR 0.553, 95% CI: 0.388–0.788, *p* = 0.001).

**Fig. 3.**
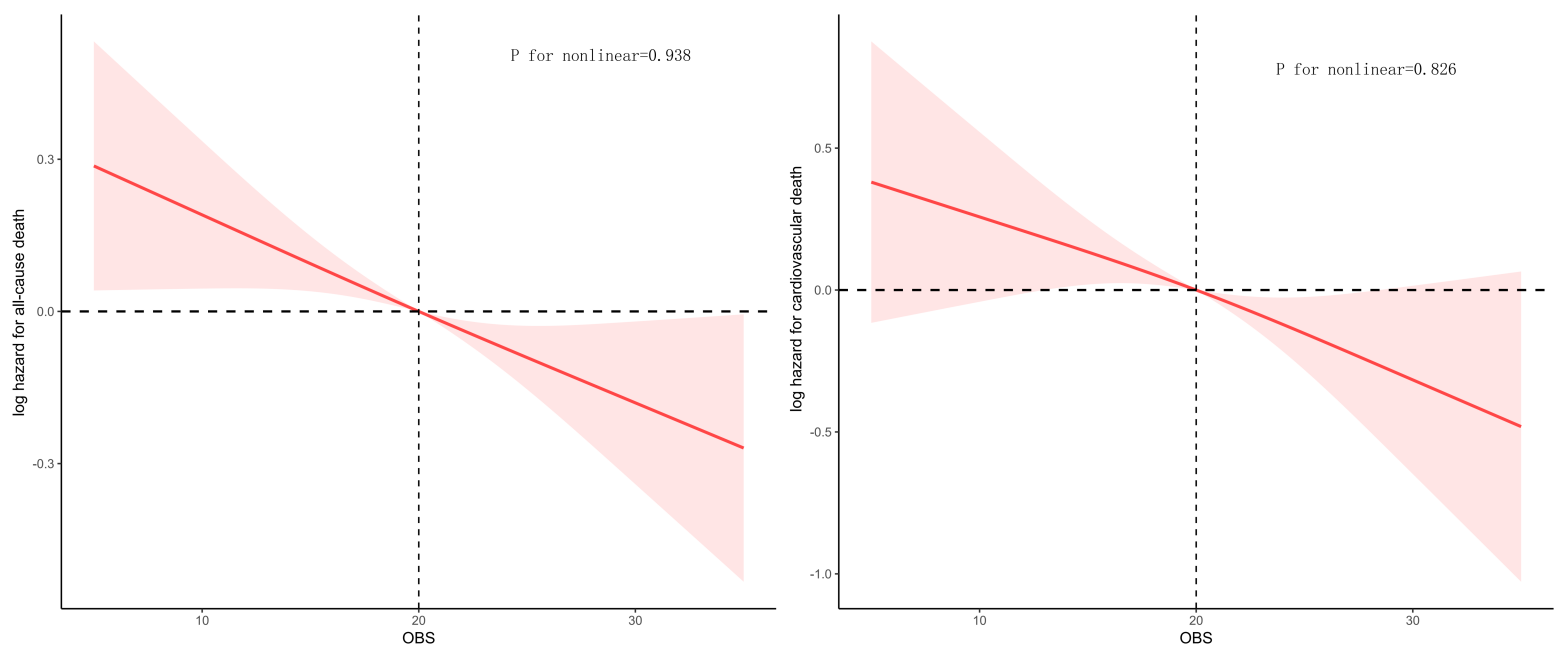
**Restricted cubic splines (RCS) analysis for mortality**.

The negative linear association between OBS and mortality risk was also confirmed using RCS analysis (Fig. 3). Mortality risk progressively declined as OBS increased, with the relationship showing no significant nonlinearity for either all-cause (*p* = 0.938) or cardiovascular diseases (*p* = 0.826). These findings support the protective, dose-dependent benefits of higher OBS on mortality.

### 3.4 Subgroup Analysis

Analysis of subgroups demonstrated that the inverse relationship between OBS and all-cause mortality was the strongest among non-Hispanic whites, individuals with a high school diploma or higher, and those free of diabetes (all *p* for interaction <0.05) (Table 3). These variations may reflect greater health literacy in more educated individuals and diminished protective effects of OBS in individuals with chronic conditions. Notably, Subgroup analyses revealed no significant interaction between OBS and cardiovascular mortality (all *p* > 0.05), pointing towards the potential for OBS to offer cardiovascular benefits widely among different populations.

**Table 3.** **Stratified analysis of OBS and mortality**.

OBS		All-cause mortality		Cardiovascular mortality	
		HR (95% CI)	*p* value	HR (95% CI)	*p* value
Age, years					
	≥60	0.985 (0.976, 0.994)	0.002	0.982 (0.964, 1.001)	0.065
	<60	0.978 (0.953, 1.003)	0.087	0.954 (0.900, 1.012)	0.123
	Interaction *p*-value		0.330		0.283
Sex					
	Male	0.987 (0.975, 1.000)	0.055	0.977 (0.954, 1.000)	0.055
	Female	0.974 (0.960, 0.988)	<0.001	0.963 (0.940, 0.986)	0.002
	Interaction *p*-value		0.223		0.288
Race					
	Non-Hispanic White	0.977 (0.966, 0.987)	<0.001	0.967 (0.948, 0.986)	0.001
	Non-Hispanic Black	1.012 (0.991, 1.033)	0.242	1.001 (0.967, 1.037)	0.912
	Mexican American	0.981 (0.952, 1.010)	0.213	0.939 (0.879, 1.003)	0.061
	Other Hispanic	0.968 (0.918, 1.021)	0.235	0.997 (0.883, 1.126)	0.970
	Other race	1.024 (0.973, 1.078)	0.357	1.057 (0.894, 1.250)	0.511
	Interaction *p*-value		0.018		0.273
Education					
	Above high school	0.971 (0.957, 0.985)	<0.001	0.971 (0.944, 0.999)	0.042
	High school or equivalent	0.998 (0.982, 1.014)	0.808	0.985 (0.956, 1.015)	0.342
	Below high school	0.980 (0.963, 0.998)	0.032	0.964 (0.933, 0.997)	0.033
	Interaction *p*-value		0.013		0.636
Marital status					
	Coupled	0.983 (0.969, 0.996)	0.016	0.973 (0.949, 0.998)	0.034
	Singled	0.983 (0.969, 0.997)	0.020	0.973 (0.950, 0.997)	0.029
	Interaction *p*-value		0.912		0.717
PIR					
	PIR >3.0	0.971 (0.952, 0.991)	0.004	0.970 (0.941, 0.999)	0.048
	PIR 1.0–3.0	0.982 (0.969, 0.995)	0.008	0.967 (0.942, 0.993)	0.013
	PIR ≤1.0	0.993 (0.969, 1.017)	0.588	0.986 (0.926, 1.050)	0.680
	Interaction *p*-value		0.348		0.862
Smoking					
	Yes	0.982 (0.969, 0.995)	0.009	0.977 (0.952, 1.002)	0.078
	No	0.980 (0.965, 0.995)	0.011	0.962 (0.940, 0.985)	0.001
	Interaction *p*-value		0.887		0.642
Drinking					
	Yes	0.980 (0.970, 0.989)	<0.001	0.969 (0.951, 0.987)	0.001
	No	0.999 (0.978, 1.021)	0.968	0.999 (0.954, 1.045)	0.966
	Interaction *p*-value		0.064		0.142
Diabetes status					
	DM	1.000 (0.983, 1.017)	0.976	0.979 (0.945, 1.015)	0.259
	Pre-DM	0.964 (0.934, 0.994)	0.020	0.935 (0.882, 0.990)	0.021
	No	0.976 (0.964, 0.987)	<0.001	0.974 (0.952, 0.998)	0.033
	Interaction *p*-value		0.045		0.498
Hyperlipidemia					
	Yes	0.979 (0.969, 0.988)	<0.001	0.968 (0.950, 0.986)	<0.001
	No	0.993 (0.969, 1.017)	0.596	0.981 (0.933, 1.032)	0.475
	Interaction *p*-value		0.370		0.383

## 4. Discussion

The study found that among participants with hypertension, there was a significant protective link between higher OBS and lower rates of both all-cause and cardiovascular mortality. Elevated OBS values reflect greater antioxidant exposure and reduced oxidative stress, which correlates with a lower risk of mortality. Subgroup analysis revealed a particularly strong inverse association for non-Hispanic whites, highly educated, and non-diabetic individuals. The study emphasizes oxidative stress as a key contributor to the progression of hypertension and related comorbidities, suggesting that an antioxidant-focused diet and lifestyle could yield better health outcomes for hypertensive patients.

Considerable research has focused on the association between oxidative stress and hypertension [19, 20, 21]. Higher OBS levels correlate with a reduced risk of developing hypertension [22], underscoring the importance of an antioxidant-rich diet and healthy lifestyle for prevention. Additionally, antioxidant supplementation has been shown to improve arterial stiffness and endothelial function in hypertensive patients [8], indicating its potential therapeutic role. Studies have also found that hypertension induced by salt and fructose is linked to inflammatory responses triggered by ROS and free radicals [23], while antioxidant-rich substances such as green tea can effectively lower blood pressure and improve cardiovascular function [24]. Dietary nitrates have been shown to alleviate oxidative stress and inflammation, and traditional herbal formulas, such as Tianma Gouteng Decoction, have demonstrated the ability to reverse hypertension-related cardiovascular remodeling by regulating oxidative stress and inflammation [25]. Collectively, these findings suggest that oxidative stress is a crucial driver of hypertension pathology and a potential treatment target. Improving outcomes may be possible by regulating oxidative balance.

At the molecular and cellular levels, oxidative stress plays a vital role in initiating and progression of cardiovascular diseases. Excessive production of free radicals, particularly ROS, leads to lipid peroxidation, protein modification, and DNA damage, which trigger inflammatory responses and cell apoptosis [26]. For example, oxidized low-density lipoprotein (ox-LDL) is a key contributor to early atherosclerosis, promoting arterial plaque formation by increasing macrophage uptake and foam cell formation [27]. Oxidative stress also contributes to endothelial dysfunction and vasoconstriction through the inactivation of tetrahydrobiopterin (BH4), leading to dysregulation of endothelial nitric oxide synthase (eNOS) [28]. Furthermore, recent studies have shown that oxidative stress activates multiple inflammatory pathways, including NF-κB, the NOD-like receptor protein 3 (NLRP3) inflammasome, and NADPH oxidase (NOX) [29, 30]. These pathways intensify vascular inflammation and accelerate the progression of atherosclerosis, further underscoring the detrimental impact of oxidative stress in cardiovascular pathology [31, 32].

Individuals with higher OBS scores typically exhibit greater intake of dietary antioxidants, including polyphenols, flavonoids, and vitamins C and E, which can directly or indirectly neutralize free radicals and inhibit inflammatory pathways related to oxidative stress. Additionally, those with higher OBS are likely to have lower exposure to pro-oxidative factors such as smoking, alcohol use, and high-fat diets, which may contribute to the observed reduction in mortality risk. Unlike conventional biomarkers such as thiobarbituric acid-reactive substances (TBARS), superoxide dismutase (SOD), and glutathione peroxidase (GSHPx)—which reflect short-term oxidative changes, require invasive sampling, and are difficult to standardize in large populations—OBS offers a non-invasive, stable, and integrative measure of long-term oxidative balance. By combining dietary and lifestyle factors, OBS captures cumulative oxidative stress exposure, making it a more practical and informative tool for studying chronic diseases like hypertension in large-scale epidemiology.

Consistent with prior investigations, this study reinforces the adverse cardiovascular effects of oxidative stress and suggests that a higher OBS may offer protective benefits for individuals with hypertension. This study also highlights that this protective effect is more pronounced in certain subgroups, emphasizing the critical role of antioxidant status in the prognosis of hypertension patients.

Several limitations should be taken into consideration in this study. First, the OBS is derived from self-reported dietary and lifestyle data, which may not accurately reflect long-term changes. Second, the observational design limits the ability to establish a causal relationship between OBS and mortality outcomes. Third, the generalizability of the findings may be restricted, as the cohort was drawn from a U.S. representative sample, which may not apply to populations with differing dietary habits, healthcare systems, or genetic backgrounds. Fourth, while OBS measures oxidative balance, it may not fully encompass all sources of oxidative stress or the complex biological interactions among various antioxidant components. Furthermore, the exclusion of participants with missing critical variables could introduce selection bias, thereby compromising the representativeness of the final analytic sample and potentially resulting in either an underestimation or overestimation of the observed associations.

Despite these limitations, the investigation underscores the potential for oxidative balance to reduce mortality risk among hypertensive patients. Future research should focus on longitudinal studies to track the long-term health effects of OBS, as well as intervention trials to determine whether lifestyle modifications can raise OBS and improve clinical outcomes. Additionally, further exploration of the underlying biological processes is warranted.

## 5. Conclusions

In conclusion, this study demonstrates an association between higher OBS levels and reduced all-cause and cardiovascular mortality among adults with hypertension. These findings underscore the importance of maintaining oxidative balance as a potential component for hypertension management and highlight the possible benefits of an antioxidant-rich dietary lifestyle for individuals living with this condition.

## Data Availability

This study’s data are publicly available at the National Center for Health Statistics (https://wwwn.cdc.gov/nchs/nhanes).

## Abbreviations

OBS, Oxidative Balance Score; NHANES, National Health and Nutrition Examination Survey; NCHS, National Center for Health Statistics; PIR, poverty-to-income ratio; K-M, Kaplan-Meier; RCS, restricted cubic splines; ROS, reactive oxygen species; ox-LDL, oxidized low-density lipoprotein; BH4, tetrahydrobiopterin; eNOS, endothelial nitric oxide synthase; NOX, NADPH oxidase; TBARS, thiobarbituric acid-reactive substances; SOD, superoxide dismutase; GSHPx, glutathione peroxidase.

## Supplementary Material

Supplementary material associated with this article can be found, in the online version, at https://doi.org/10.31083/RCM37415.
