# Supplementary material for: Association of Oxidative Balance Score With All-Cause and Cardiovascular Mortality Among Hypertensive Adults
Source: Rev Cardiovasc Med. 2025 Aug 15;26(8):37415. doi: 10.31083/RCM37415 (PMC12415749; doi:10.31083/RCM37415)
Supplement: Supplementary file 1 [file 2153-8174-26-8-37415-s1.zip › Supplementary Table 1. Oxidative balance score..docx]

**Table S1. Oxidative balance score.**

| OBS components | Property | Female | | | Male | | | |
| --- | --- | --- | --- | --- | --- | --- | --- | --- |
|  |  | 0 | 1 | 2 | 0 | 1 | 2 |  |
| **Dietary OBS** |  |  |  |  |  |  |  |  |
| Dietary fiber(g/d) | A | ＜11.20 | 11.2-16.70 | ≥16.70 | ＜13.05 | 13.05-20.40 | ≥20.40 |  |
| Carotene (RE/d) | A | ＜60.52 | 60.52-205.60 | ≥205.60 | ＜55.87 | 55.87-183.17 | ≥183.17 |  |
| Vitamin B6(mg/d) | A | ＜1.26 | 1.26-1.83 | ≥1.83 | ＜1.63 | 1.63-2.43 | ≥2.43 |  |
| Vitamin B12(mcg/d) | A | ＜2.58 | 2.58-4.47 | ≥4.47 | ＜3.54 | 3.54-6.07 | ≥6.07 |  |
| Riboflavin (mg/d) | A | ＜1.39 | 1.39-1.99 | ≥1.99 | ＜1.75 | 1.75- 2.53 | ≥2.53 |  |
| Total Folate (mcg/d) | A | ＜256.50 | 256.50-378.50 | ≥378.50 | ＜317.50 | 317.50-479.33 | ≥479.33 |  |
| Niacin (mg/d) | A | ＜15.60 | 15.60-22.36 | ≥22.36 | ＜21.08 | 21.08-30.41 | ≥30.41 |  |
| Vitamin C (mg/d) | A | ＜44.25 | 44.25-95.95 | ≥95.95 | ＜44.15 | 44.15-102.95 | ≥102.95 |  |
| Vitamin E (ATE) (mg/d) | A | ＜4.67 | 4.67-7.51 | ≥7.51 | ＜5.67 | 5.67-8.85 | ≥8.85 |  |
| Calcium (mg/d) | A | ＜577.0 | 577.0- 875.50 | ≥875.50 | ＜677.50 | 677.50-1049.00 | ≥1049.00 |  |
| Magnesium (mg/d) | A | ＜200.50 | 200.50-280.5 0 | ≥280.5 0 | ＜248.00 | 248.00-348.00 | ≥348.00 |  |
| Zinc (mg/d) | A | ＜7.06 | 7.06-10.31 | ≥10.31 | ＜9.45 | 9.45-14.00 | ≥14.00 |  |
| Copper (mg/d) | A | ＜0.85 | 0.85-1.20 | ≥1.20 | ＜1.04 | 1.04-1.47 | ≥1.47 |  |
| Selenium (mcg/d) | A | ＜71.85 | 71.85-102.40 | ≥102.40 | ＜95.80 | 95.80-135.68 | ≥135.68 |  |
| Iron (mg/d) | P | ≥14.18 | 9.79-14.18 | ＜9.79 | ≥18.15 | 12.34-18.15 | ＜12.34 |  |
| Total fat (gm/d) | P | ≥73.13 | 48.75-73.13 | ＜48.75 | ≥96.73 | 65.46-96.73 | ＜65.46 |  |
| **Lifestyle OBS** |  |  |  |  |  |  |  |  |
| Physical activity  (MET-minutes/week) | A | ＜480.00 | 480.00-1680.00 | ≥1680.00 | ＜600.00 | 600.00-2480.00 | ≥2480.00 |  |
| cotinine(ng/mL) | P | ≥0.092 | 0.017-0.092 | ＜0.017 | ≥0.391 | 0.023-0.391 | ＜0.023 |  |
| Alcohol(g/d) | P | ≥15 | (0, 15) | non | ≥30 | (0, 30) | non |  |
| Body mass index(kg/m2) | P | ≥30 | [25, 30) | ＜25 | ≥30 | [25, 30) | ＜25 |  |

OBS: oxidative balance score; A: antioxidant; P: prooxidant; RE: retinol equivalent; ATE: alpha-tocopherol equivalent; MET: metabolic equivalent.
